# Environmental Surveillance of Polioviruses in Armenia, Colombia before Trivalent Oral Polio Vaccine Cessation

**DOI:** 10.3390/v11090775

**Published:** 2019-08-23

**Authors:** María Mercedes González, Magile C. Fonseca, Carlos Andrés Rodríguez, Alejandra María Giraldo, José Joaquín Vila, Jhon Carlos Castaño, Leonardo Padilla, Luis Sarmiento

**Affiliations:** 1 Center of Biomedical Research, Faculty of Health Sciences, Universidad del Quindío, Armenia 630003, Colombia; 2 Enterovirus Laboratory, Department of Virology, Pedro Kourí Institute of Tropical Medicine, Havana 11400, Cuba; 3 Department of Clinical Sciences, Skåne University Hospital, Lund University, Malmo 21428, Sweden

**Keywords:** environmental surveillance, Sabin polio virus, enterovirus

## Abstract

Although acute flaccid paralysis (AFP) surveillance is the “gold standard” for detecting cases of polio, environmental surveillance can provide supplementary information in the absence of paralytic poliomyelitis cases. This study aimed to detect the introduction and/or circulation of wild poliovirus or vaccine-derived polioviruses (VDPV) in wastewater, covering a significant population of Armenia, Colombia, before trivalent oral polio vaccine (OPV) cessation. Between March and September 2015, 24 wastewater samples were collected from eight study sites in eight communes of Armenia, Colombia. Virus detection and characterization were performed using both cell culture (i.e., RD or L20B cells) and RT-PCR. Polioviruses were isolated in 11 (45.8%) of 24 wastewater samples. All isolates were identified as Sabin strains (type 1 = 9, type 3 = 2) by intratypic differentiation. Type 2 poliovirus was not detected in any of the samples. No wild poliovirus or VDPV was detected among the isolates. Non-polio enterovirus was identified in 8.3% (2/24) of the samples. This study revealed the excretion of Sabin poliovirus from OPV-immunized individuals, as well as the absence of VDPV and wild poliovirus in wastewaters of Armenia, Colombia. This confirms that environmental surveillance is an effective method, as an additional support to AFP surveillance, to monitor poliovirus during the OPV-to-IPV (inactivated polio vaccine) transition period.

## 1. Introduction

In 2012, the World Health Assembly declared the completion of poliomyelitis eradication as a programmatic emergency for global public health [1]. In response to this, the World Health Organization (WHO) executive council approved the goals, objectives, and chronogram of the Polio Eradication and Endgame Strategic Plan 2013–2018 [2]. The main implementation components of this initiative are: (1) the detection and interruption of the poliovirus transmission and maintenance of acute flaccid paralysis (AFP) surveillance in individuals <15 years of age; (2) the global switch from trivalent oral polio vaccine (tOPV) to bivalent oral polio vaccine (bOPV, types 1 and 3) along with at least one dose of “killed” or inactivated polio vaccine (IPV) for risk mitigation; and (3) the implementation of poliovirus containment measures to minimize the risk that the virus could enter the environment and cause harm.

Within this context, strengthening epidemiological surveillance to rapidly detect cases is crucial to monitoring the progress of poliomyelitis eradication. AFP surveillance is the primary mechanism for detecting poliovirus in a population [3]; however, surveillance programs in Colombia face similar barriers to those reported in some regions of Latin America, which tend to focus on cases confirmed by clinical criteria only. This may lead to sub-notification and underestimation of polio cases [4]. Under these circumstances, environmental surveillance of poliovirus plays a key role in monitoring the importation of wild type poliovirus and the detection of emerging vaccine-derived polioviruses (VDPV).

Environmental surveillance includes the control of wastewater or other environmental samples to verify the presence of poliovirus. Such surveillance relies on the fact that poliovirus replicates in intestinal lymphatic tissue and is excreted through feces to the environment, in the presence or absence of clinical symptoms [5]. It has been shown that VDPV and wild poliovirus may remain infectious for up to two months in wastewater, depending upon environmental factors [6,7]. Detection of poliovirus in the wastewater of the local community reflects the presence of virus-shedding individuals. Thereby, environmental surveillance complements clinical AFP surveillance for possible polio cases [8]. Experiences in many countries, including Finland, Egypt, India, and Israel, have confirmed that environmental surveillance can detect the introduction of wild poliovirus or VDPV before the appearance of AFP cases [7].

In 2005, environmental surveillance was conducted for the first time in the department of Quindío, Colombia in order to track the poliovirus´s circulation within communities. This study demonstrated the absence of VDPV or wild poliovirus in the analyzed region [9]. Ten years after this study, the expanded program of immunizations in Colombia was proposed to enhance the vaccination program by introducing IPV for routine immunization, replacing the live oral polio vaccine (OPV). These changes highlight the importance of strengthening environmental surveillance programs to monitor the importation of wild type poliovirus and the detection of emerging VDPVs before the cessation of OPV.

## 2. Material and Methods

### 2.1. Type of Study and Locations

To investigate the prevalence of poliovirus in municipal wastewater samples, a cross-sectional exploratory study was performed in Armenia, Colombia between March and September 2015. Armenia, the capital of the department of Quindío, is the third most populated city in Colombia’s coffee axis. The National Administrative Department of Statistics (DANE) has estimated Armenia’s population at 290,480 inhabitants, of which 97.2% live in the urban zone. The 282,565 inhabitants located in the urban area are distributed across 10 communes. In total, eight sampling sites across eight communes were selected, according to the indications provided by the regional Water and Sewage Company. The selection of the sampling sites was made on the basis of population size, accessibility, immunization coverage, sanitation, proximity to industrial areas to avoid mixture with industrial waste or agricultural runoff, and presence of converging sewer lines that receive wastewater from a considerable proportion of the population in the catchment area. Each selected commune has at least one health center where the OPV was administered, according to the national immunization schedule approved by the Colombian Ministry of Health. The vaccination schedule consisted of three doses given at 2, 4, and 6 months of age, and as a booster dose at 18 months and 5 years of age. In 2015, OPV vaccine coverage in Armenia, Quindío was estimated at 95% [10].

### 2.2. Wastewater Sampling

A total of 24 raw sewages were collected at the main sewage line that enters the wastewater treatment facility (WWTP) serving the selected commune’s urban areas, using the grab method as described in the WHO Guidelines for Environmental Surveillance of Poliovirus [11]. Raw sewage was usually taken once a month, at each of the eight sampling sites, at different scheduled times during the peak hours of household sewage flow (i.e., between 08.30 a.m. and 11.30 a.m.) to make a composite sample with a total volume of 1 liter. A summary of environmental surveillance, by sampling site, during the 7 month sampling in Armenia, Colombia in 2015 is presented in Table 1. The sampling was conducted according to the procedures of good practices to either protect the person in charge of taking the samples or prevent cross-contamination between samples. The wastewater samples were transported in clean, leakproof containers at 4 °C to the Biomedical Research Center at the University of Quindío.

### 2.3. Wastewater Preparation

Viral concentration was performed according to the method reported by Sobsey et al. [12] and described elsewhere [6]. In brief, the wastewater sample was centrifuged at 4 °C for 30 minutes at 5000 × *g* to sediment (pellet) the sewage solids. The supernatant was recovered for further processing. The solids were then re-suspended in five volumes of elution medium (3% beef extract), and one half volume of chloroform. This mixture was centrifuged at 4 °C for 30 minutes at 5000 × *g* to sediment the chloroform and sewage solids, and the aqueous supernatant was recovered. The chloroform and sewage solids were supplemented with another volume of elution medium, the mixture was re-extracted and centrifuged, and the resulting aqueous supernatant was recovered. The supernatant extracts from the sewage solids were combined with the original sewage supernatant, and viruses were precipitated from the mixture with polyethylene glycol 8% (PEG) and sodium chloride 0.3 M (NaCl) at 4 °C overnight. The precipitated viruses were sedimented by centrifugation at 5000 × *g* for 1 hour at 4 °C. The resulting sediment was re-suspended in 2 mL of PBS and the mixture was extracted with chloroform. After centrifuging to remove the chloroform, the aqueous supernatant was recovered and the remaining chloroform was re-extracted with a volume of elution medium. The elution medium was recovered and combined with the previously collected aqueous supernatant. After treatment, 6 mL of sample was obtained, resulting in an approximately 150-fold volume reduction.

To evaluate the efficiency of virus recovery from wastewater, 2 × 10^4^ 50% cell culture infectious doses (CCID_50_) of poliovirus type 1 Sabin strains were mixed into a selected sewage sample that was unlikely to inherently contain poliovirus. The sewage sample was autoclaved at 121 °C for 30 min to inactivate any virus possibly present in the sample. The mixture was concentrated, as described above, in parallel with a homologous non-spiked sample. The concentrates were titrated by the terminal dilution method in Hep2 C cells, as recommended by the WHO [13]. The recovery of spiked poliovirus was 25 and 32% in two independent experiments.

### 2.4. Viral Isolation

**Figure 1:**
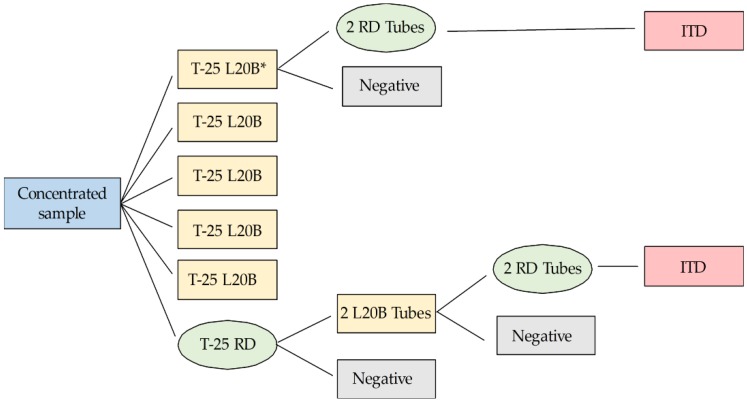
Flowchart of poliovirus isolation protocol. This is an alternative test algorithm for poliovirus isolation and characterization, which is an adaptation of the World Health Organization (WHO) guidelines for environmental surveillance of poliovirus circulation [14]. *All five 25 cm^2^ flasks containing monolayers of L20B cells (that were not to be pooled for freezing) followed the same path. Tubes were pooled together.

Viral isolation was carried out according to the conditions and algorithm suggested by the WHO [13,14]. A 0.5 mL volume of each concentrate sample was inoculated onto five 25 cm^2^ flasks containing monolayers of L20B cells and one 25 cm^2^ flask containing RD cells. L20B cells (a mouse L cell line stably transfected with the human receptor for poliovirus, National Institute for Biological Standards and Control, NIBSC) are especially permissive to poliovirus growth and excluding of most other species of enterovirus. RD cells (derived from human rhabdomyosarcoma tumor cells, American Type Culture Collection (ATCC) CCL-136™) are susceptible to most human enteroviruses, including poliovirus. Cell line stocks were derived from cultures provided by the Institute of Tropical Medicine in Havana, Cuba. The cultures were incubated at 37 °C and the cytopathic effect (CPE) was monitored daily, for five days, by microscopic examination. Samples showing CPE in the L20B flasks were frozen and thawed for passage onto two tubes with fresh RD monolayers. If the RD tubes were positive, possible poliovirus was reported in the L20B flasks. On the other hand, any positive flask of RD was passed into two tubes with fresh monolayer cultures of L20B cells. If these L20B tubes were positive, L20B cell isolates were cross-passaged on two tubes with fresh monolayer cultures of RD cells. Any sample showing CPE in RD, from cross-passaged L20B isolates, was classified as “suspected poliovirus”. Any culture negative in L20B but positive in RD was classified as “non-polio enterovirus”, as seen in Figure 1.

### 2.5. Identification and Characterization of the Isolate

The presence of enterovirus in cell culture positive samples was determined by reverse transcription-polymerase chain reaction (RT-PCR) assay, with primer pair matching highly conserved sites within the 5′-non-coding regions of enterovirus genomes. Poliovirus isolates were identified using pan-poliovirus and serotype-specific primers. Confirmed polioviruses were further characterized as Sabin or wild strains with Sabin type 1, 2, and 3 specific primers. The primers used in this study are summarized in Table 2 [15,16,17,18]. Sabin isolates were submitted to Colombia’s National Health Institute and the Center for Disease Control (CDC) for screening by the real-time VDPV assay, as described elsewhere [19]. Isolates showing concordant results between the intratypic differentiation (ITD) PCR and VDPV screening were classified as Sabin strains.

### 2.6. Ethics Statement

The study was conducted in compliance with the principles expressed in the Declaration of Helsinki and in the European Council’s Convention on Human Rights and Biomedicine. All methods were carried out in accordance with relevant guidelines and regulations. This study did not involve human participants or human experimentation; the only human materials used were wastewater samples collected from sewer effluent.

## 3. Results

### 3.1. Environmental Virus Isolations

From the 24 samples of wastewater collected at all sites, 13 (54.1%) were positive on at least one cell line. Eleven of the 13 isolates (45.8%) showed CPE characteristics of enterovirus in RD and L20B cells, while two samples (8.3%) showed CPE only in the RD line (Table 3). All non-polio enteroviruses were isolated only on RD cells, while poliovirus strains were isolated on RD and L20B cells. The L20B cells were only able to isolate poliovirus.

### 3.2. Identification and Characterization of Isolated Polioviruses

Of the 11 positive samples in both cell lines, type 1 poliovirus was identified in nine samples and type 3 poliovirus in two samples. Type 2 poliovirus was not detected in any of the samples. Viruses identified as type 1 poliovirus and type 3 poliovirus were characterized as Sabin type 1 poliovirus and Sabin type 3 poliovirus. There were no discrepancies between the ITD and VDPV screening, thus ruling out the presence of VDPV in the environmental samples examined during the study period. Wild polioviruses were not detected among the isolates. The two isolates that were positive in RD and negative in L20B were characterized as non-polio enterovirus through RT-PCR using generic primers from the 5′-end non-coding region of the enterovirus. Results were confirmed by the CDC in Atlanta, USA.

## 4. Discussion

This study evaluated the possible presence of VDPVs, wild type polioviruses, and other enteroviruses during sewage surveillance when OPV was used in the routine immunization program in Armenia, Colombia. Sabin poliovirus type 1 and type 3 were successfully isolated from OPV-immunized individuals. No wild poliovirus or VDPV was isolated from the environmental samples examined during the study period. Remarkably, Sabin strains were isolated in approximately 50% of the grab samples, proving this method to be an effective supplemental support to AFP surveillance to sensitively monitor poliovirus (wild, VDPV, and Sabin) within a population [7]. Our results appear to be consistent with those of a previous study conducted by our laboratory, which led to the demonstration of an absence of VDPV and wild poliovirus in Colombian departments with vaccination coverage below 80% [20]. This implies the effectiveness of polio immunization and a high level of protection against polio in the region.

The present study used a WHO international standard method for virus collection, recovery, and concentration that was developed in a similar study in the Quindío department more than 10 years ago [9], wherein wastewater samples were collected from the same sampling sites as those selected in 2015. In agreement with the present study, the poliovirus isolates in 2005 were identified as Sabin poliovirus type 1 and type 3. Interestingly, a mixture of types 1 and 3 Sabin poliovirus vaccine was identified in five samples that tested positive for poliovirus [9]; however, unlike the prior study, we did not find a poliovirus mixture in any of the 11 samples that tested positive for poliovirus. This is an important finding for the understanding of how the circulation of poliovirus behaves 10 years after the first study, and it confirms that local vaccine coverage has been able to maintain an area free of wild poliovirus and VDPV.

In both studies, type 2 poliovirus was not identified in any of the samples. These findings confirm the results of previous studies conducted in Cuba, which showed that percentages of type 2 poliovirus in wastewater are lower than those found for types 1 and 3 [6,21]. This observation is consistent with the lower rate of type 2 poliovirus isolation in stools randomly sampled from the population of the area where wastewater was collected, indicating the lower excretion of this serotype in relation to types 1 and 3 [6,21]. Furthermore, it has been demonstrated that Sabin 2 is considerably more immunogenic than types 1 or 3 [20]. It is likely that a higher immunological response to the type 2 component of the OPV limits the excretion of this serotype in stool and, consequently, there was lack of Sabin type 2 strains in wastewater. In contrast, other studies have shown the presence of type 2 strains in the environment [22,23,24,25]. It is important to note that the recovery rates of our approach were found to be comparable to those of the two-phase separation method, which is the WHO-recommended concentration/separation method that has been extensively and effectively used for poliovirus environmental surveillance. This suggests that the lack of type 2 strains in the present study was not due to less efficient poliovirus recovery. Additionally, non-polio enterovirus isolation in the present study can be considered a proxy indicator to validate that the environmental samples were processed and analyzed appropriately to preserve infectiveness [5]. This, in turn, confirms previous results showing that non-polio enterovirus circulates broadly in the region [20,21,22,23,24,25,26].

This study has limitations and the results should be interpreted with caution. Our conclusions are based mainly on the environmental surveillance of poliovirus in urban sewage, in the capital of a department of Colombia; therefore, our results may not be generalizable to the whole country or departments with different wastewater collection systems or different vaccination coverages. Since Colombia has become a transit zone for migrants and displaced peoples, future studies should aim to replicate results in Colombian regions with low vaccination coverage and migrant traffic. Implementing an effective environmental surveillance system in these areas is essential for the early detection of possible reintroduction and transmission of poliovirus in Colombia and, thus, preventive measures against the possible appearance of poliomyelitis outbreaks during the OPV-to-IPV transition period are needed. A further limitation of our study is that the rate of usage of disposable diapers containing stool from OPV-immunized children in the catchment communes is unknown; therefore, if disposable diapers containing stool from OPV-immunized children were properly treated and discarded, the sewage samples from this study may not reflect the excretion content from children wearing diapers.

Overall, our results demonstrate the excretion of Sabin poliovirus from OPV-immunized individuals, and show the absence of VDPV and wild poliovirus in Armenia, Colombia. This confirms the usefulness of environmental surveillance to monitor fecal shedding of poliovirus from large populations connected to sewer or drainage systems, and provides valuable information, at the local community level, on the circulation of poliovirus before OPV cessation.

## Figures and Tables

**Table 1:** Sampling per month for each collector and district during the environmental surveillance in Armenia, Colombia in 2015.

Communes	Collector SewerLocation	No. Inhabitants in Catchment Area	No. of Samples/Month (March to September)							Total
			M	A	M	J	J	A	S	
Quimbaya	Aldana	46,614	1		1		1		1	4
El Cafetero	C. Diablo	37,808	1			1			1	3
Rufino José Cuervo Sur	Sta Rita	49,224		1		1		1	1	4
Centenario	Pinares	33,111	1		1		1			3
Rufino José Cuervo Sur	Cristales	49,224				1		1		2
Francisco de Paula Santander	Miraflores	14,429	1			1			1	3
Alfonso López	M Beltran	33,796		1	1		1			3
Rufino José Cuervo Sur	Los Quindos	49,224		1				1		2
All										24

**Table 2:** Primers used for polymerase chain reaction (PCR) amplification and identification.

Primers	Sequences
Generic enterovirus	EVS 5′-CTCCGGCCCCTGAATGCGGCT A-3′ EVA 5′-ATTGTCACCATAAGCAGCC-3′
Group of polioviruses	Pan PV S 5′-TTIAIIGC(A/G)TGICC(A/G)TT(A/G)TT-3′ Pan PVA 5′-CITAITCI(A/C)GITT(C/T)GA(C/T)ATG-3′
Poliovirus type 1	PV1 2S 5′-TGCGIGA(C/T)ACIACICA(C/T)AT-3′ PV1 A 5′-ATCATICT(C/T)TCIA(A/G)CAT(C/T)TG-3′
Poliovirus type 2	PV 2S 5′-TGCGIGA(C/T)ACIACICA(C/T)AT-3′ PV2 A 5′-A(C/T)ICC(C/T)TCIACI(A/G)CICC(C/T)TC-3′
Poliovirus type 3	PV3 S 5′-AA(C/T)CCITCI(A/G)TITT(C/T)TA(C/T)AC-3′ PV3 A, 5′-CCIAI(C/T)TGITC(A/G)TTIG(C/T)(A/G)TC-3′
Sabin type 1	Sabin 1R 5′-TCCACTGGCITCAGTGTT-3′ Sabin 1S 5′-AGGTCAGATGCTTGAAAGC-3′
Sabin type 2	Sabin 2A 5′-CGGCTTGTGTCCAGGC-3′ Sabin 2S 5′-CCGTTGAAGGGATTACTAAA-3′
Sabin type 3	Sabin 3A 5′-TAAGCTATCCTGTTGCC-3′ Sabin 3S 5′-AGGGCGCCCTAACIYTG-3′

**Table 3:** Poliovirus and non-polio enterovirus isolated from wastewater samples in the municipality of Armenia, Colombia (March to September, 2015).

Date	Location	Isolation		RT-PCR								Intratypic Differentiation (ITD)
		RD	L20B	EV	PV	P1	P2	P3	S1	S2	S3	
May	Aldana	+	+	+	+	+	-	-	+	-	-	Sabin poliovirus 1
Sept	Aldana	+	-	+	N/A	N/A	N/A	N/A	N/A	N/A	N/A	Non-polio enterovirus
June	C. Diablo	+	+	+	+	+	-	-	+	-	-	Sabin poliovirus 1
April	Sta Rita	+	+	+	+	+	-	-	+	-	-	Sabin poliovirus 1
Sept	Sta Rita	+	+	+	+	+	-	-	+	-	-	Sabin poliovirus 1
June	Cristales	+	+	+	+	+	-	-	+	-	-	Sabin poliovirus 1
March	Miraflores	+	+	+	+	+	-	-	+	-	-	Sabin poliovirus 1
June	Miraflores	+	+	+	+	+	-	-	+	-	-	Sabin poliovirus 1
Sept	Miraflores	+	+	+	+	+	-	-	+	-	-	Sabin poliovirus 1
April	Y.M. Beltran	+	+	+	+	-	-	+	-	-	+	Sabin poliovirus 3
May	Y.M. Beltran	+	+	+	+	-	-	+	-	-	+	Sabin poliovirus 3
July	Y.M. Beltran	+	+	+	+	+	-	-	+	-	-	Sabin poliovirus 1
Aug	Los Quindios	+	-	+	N/A	N/A	N/A	N/A	N/A	N/A	N/A	Non-polio enterovirus

N/A: not applicable, EV: generic primers, PV: specific primers from the poliovirus group, P1: polio serotype 1 specific primers, P2: polio serotype 2 specific primers, P3: polio serotype 3 specific primers, S1: specific primers of Sabin 1 vaccine strains, S2: specific primers of Sabin 2 vaccine strains, S3: specific primers of Sabin 3 vaccine strains.
